# Association between ischemia-modified albumin (IMA) and peripheral endothelial dysfunction in rheumatoid arthritis patients

**DOI:** 10.1038/s41598-024-54641-5

**Published:** 2024-02-17

**Authors:** Gian Luca Erre, Ilaria Chessa, Stefania Bassu, Lorenzo Cavagna, Ciriaco Carru, Gianfranco Pintus, Roberta Giordo, Arduino Aleksander Mangoni, Giuseppe Damiano Sanna, Angelo Zinellu

**Affiliations:** 1 Dipartimento di Medicina, Chirurgia e Farmacia, University of Sassari, Sassari, Italy; 2 UO Reumatologia, Azienda Ospedaliero-Universitaria di Sassari, Sassari, Italy; 3 Dipartimento di Scienze Biomediche, University of Sassari, Sassari, Italy; 4 Rheumatology Division, University and IRCCS Policlinico S. Matteo Foundation, Pavia, Italy; 5 Discipline of Clinical Pharmacology, College of Medicine and Public Health, Flinders University, Adelaide, Australia; 6 Department of Clinical Pharmacology, Flinders Medical Centre, Southern Adelaide Local Health Network, Adelaide, Australia; 7 UO Cardiologia, Azienda Ospedaliero-Universitaria di Sassari, Sassari, Italy

**Keywords:** Oxidative stress, Rheumatoid arthritis, Cardiovascular disease, Pulse amplitude tonometry, Reactive oxygen species, Inflammation, Atherosclerosis, Endothelial dysfunction, Rheumatology, Immunology, Autoimmunity, Immunological disorders

## Abstract

The identification of circulating biomarkers of endothelial dysfunction (ED), a precursor to atherosclerosis, in rheumatoid arthritis (RA) would facilitate early risk stratification and prevention strategies. Ischemia-modified albumin (IMA) has emerged as a potential biomarker of oxidative stress, ischemia, and ED. However, studies examining the relationship between IMA and ED in RA patients are lacking. We measured serum IMA concentrations by using an albumin cobalt binding test and peripheral vasodilatory capacity by EndoPAT in 113 RA patients without previous cardiovascular events enrolled in the EDRA study (ClinicalTrials.gov: NCT02341066). The mean peripheral vasodilatory capacity, expressed by the log of reactive hyperemia index (logRHI), was 0.82, corresponding to 27% RA patients having ED. The mean plasma concentrations of IMA were 0.478 absorbance units. We observed a significant and inverse association between peripheral vasodilatory capacity and serum IMA concentrations (rho =  − 0.22, p = 0.02). In univariate logistic regression, ED was significantly associated with serum IMA concentrations [OR 1173 (95% CI 1.3568 to 101,364), p = 0.040) and higher disease activity. In multivariate logistic regression, the independent association between ED and IMA remained significant after correction for disease activity and other RA-confounders [OR 2252 (95% CI 1.0596 to 4,787,505), p = 0.048 in Model 1; OR 7221 (95% CI 4.1539 to 12,552,859), p = 0.02 in Model 2]. Conclusions: This study suggests that IMA is a promising biomarker of ED in RA. Further research is needed to confirm our findings and determine the clinical utility of IMA in detecting and managing early atherosclerosis in RA patients.

## Introduction

Rheumatoid arthritis (RA) is a chronic progressive disease associated with systemic inflammation that affects mainly synovial joints leading to tissue destruction, disability and excess of mortality^1^. The prevalence rate for RA in European countries is approximately 0.5–1% of the adult population. RA patients suffer of a life expectancy significantly reduced with respect to the general population (by 3 to 18 years) with a standardized mortality ratio ranging from 1.2 to 2.7^2,3^. About one-third of premature deaths in RA are due to atherosclerotic cardiovascular disease including coronary heart disease. The increased prevalence of atherosclerosis in RA seems to be associated with excess inflammatory burden and requires tailored screening strategies and management^4–7^.

Increased oxidative stress, local autoimmune response, and specific activity of pro-inflammatory cytokines have been linked to endothelial dysfunction (ED), early atherosclerosis, and increased risk of arrhythmias and other cardiovascular events in RA^8–15^.

Ischemia modified albumin (IMA), resulting from chemical changes of albumin during ischemia, has been suggested as a candidate biomarker for acute coronary syndrome^16^. Several studies have also reported increased serum IMA concentrations in non-cardiac diseases (e.g., sepsis, intestinal ischemia, acute pulmonary embolism) as a result of a common background of acute and chronic inflammatory state, increased oxidative stress, and reactive oxygen species formation^17^. Moreover, IMA formation has been related to ED, the early step of atherosclerotic vascular disease^18^.

Elevated serum IMA concentrations have also been reported in RA patients compared to the general population and have been linked to inflammatory state, oxidative stress, and increased carotid intima-media thickness^19–21^. However, the possible link between IMA and ED in this patient group has not been investigated.

Therefore, we sought to address this issue by studying the relationship between serum IMA concentrations and disease activity and peripheral endothelial vasodilatory capacity in RA patients free of previous cardiovascular events.

## Materials and methods

### Patients

Consecutive unselected RA patients fulfilling the 2010 European League Against Rheumatism and American College of Rheumatology classification criteria^22^ were prospectively enrolled in the Bio-RA study between October 2015 and July 2017. The Bio-RA study is an ancillary study of the Endothelial Dysfunction Evaluation for Coronary Heart Disease Risk Estimation in Rheumatoid Arthritis study (ClinicalTrials.gov: NCT02341066). Inclusion and exclusion criteria for both studies have been previously reported^9,12^.

All participants in the Bio-RA and EDRA studies underwent a comprehensive assessment of traditional cardiovascular risk factors, including hypertension (defined as a blood pressure ≥ 140/90 mmHg or treatment with antihypertensive medications), diabetes (diagnosed according to the patient’s history and/or treatment with insulin or oral hypoglycemic agents), dyslipidemia (defined according to either a recent lipid profile, the patient’s history and/or treatment with hypolipidemic drugs), and smoking status. Additionally, data on current treatment with disease-modifying antirheumatic drugs (DMARDs) as well as laboratory parameters of inflammation, such as C-reactive protein (CRP), erythrocyte sedimentation rate (ESR), and neutrophil-to-lymphocyte ratio (NLR)^23,24^, Disease Activity Score-28 (DAS-28), positivity for Rheumatoid Factor (RF), and anticitrullinated cyclic peptide antibodies (ACPA), were collected. RA patients with DAS-28 ≤ 2.6 were considered in remission, while those with DAS-28˃2.6 were considered as having active disease.

### Pulse amplitude tonometry

Peripheral vasodilatory capacity was assessed using a non-invasive and validated technique known as pulse amplitude tonometry (PAT) measured using the EndoPAT 2000 system (Itamar Medical Inc., Caesarea, Israel). EndoPAT is an FDA-approved device that measures changes in peripheral arterial tone and pulsatile blood flow in response to reactive hyperemia. This technique is based on the principle that increased blood flow through an occluded artery leads to an increase in shear stress, which induces endothelium-dependent vasodilation. EndoPAT measures this response by assessing the ratio of pulse amplitude after reactive hyperemia to the baseline pulse amplitude. A reactive hyperemia index (RHI) is calculated as the ratio of the post-occlusion pulse amplitude to the baseline pulse amplitude, and the logarithmic transformation of this index (lnRHI) is used as a marker of peripheral vasodilatory capacity with values of lnRHI ≤ 0.51 used as a marker of ED^25^.

### Ischemia-modified albumin

Blood samples were collected using blood evacuation tubes containing EDTA (Vacutainer Systems Europe; Becton Dickinson, Meylan Cedex, France), on the same day of endothelial function evaluation and before EndoPAT testing.

Ischemia-modified albumin (IMA) was measured using an albumin cobalt binding test. 50 μL of 0.1% cobalt (II) chloride (CoCl2, 6H2O) was added to the patient’s serum and incubated for 10 min. Then, 50 μL of 1.5 mg/mL dithiothreitol was added and incubated for 2 min after the mixing procedure. Finally, 1 mL of 0.9% NaCl solution was added to stop the reaction. A blank solution was prepared with the same method except for using distilled water instead of dithiothreitol. The absorbance of the samples was measured spectrophotometrically at 470 nm and results were expressed as absorbance units (ABSU).

### Statistical analysis

Data are expressed as mean values (mean ± SD) or median values (median and IQR). The Kolmogorov–Smirnov test was performed to evaluate the variable distribution. Between-group differences of continuous variables were compared using unpaired Student’s t-test or Mann–Whitney rank sum test, as appropriate. Kruskal–Wallis t-test has been used to evaluated difference in the IMA concentration across categories of increasing RA disease activity. Correlation analyses were performed by Pearson correlation test or Spearman’s correlation rank test, as appropriate.

Predictors of ED were evaluated by univariate and multivariate logistic regression analysis. Multivariate analysis included confounders with p-values of < 0.2 in univariate analysis (DAS-28, ESR, NLR, ACPA and IMA). In case of collinearity between confounders, separate models were built by considering separate variables in each model.

This is a pilot study, and a formal sample size analysis has not been performed.

Statistical analyses were performed using MedCalc for Windows, version 19.4.1 64-bit (MedCalc Software, Ostend, Belgium).

### Institutional review board statement

The Bio-RA and the EDRA studies were approved by the Ethics Committee of Azienda ASL 1 of Sassari (Italy) (2126/CE-2015 and 2219/CE-2015) and conducted in accordance with the Declaration of Helsinki.

### Informed consent statement

Informed consent was obtained from all subjects involved in the study.

## Results

### Patients

We enrolled 113 RA patients free of previous cardiovascular events (Table 1) participating to the EDRA study. Most of RA patients were middle-aged [(54.9 (50.1–59.3) years] females, with long-standing [108 (48–192) months of disease duration] and moderately active [DAS-28 = 3.76 (2.80–4.51)] disease (Table 1). In this study consecutive unselected RA patients were enrolled.

**Table 1:** Demographic, clinical and laboratory characteristics of RA patients.

	RA patients (n = 113)	Correlations with IMA	
		Rho	p-value
Age, years	54.9 (50.1–59.3)	− 0.046	0.63
Female gender, n (%)	72 (63.7)	0.098	0.30
BMI, kg/m^2^	24.5 (22.6–27.6)	0.072	0.46
Smoke, n (%)	67 (59.2)	− 0.045	0.64
Dyslipidaemia, n (%)	27 (23.8)	0.006	0.95
Hypertension, n (%)	31 (27.4)	0.038	0.69
Diabetes, n (%)	6 (5.3)	− 0.028	0.77
Steroid use, n (%)	39 (34.6)	0.102	0.28
Metotrexate use, n (%)	65 (57.5)	0.035	0.71
DMARDs use, n (no/yes)	79 (69.9)	− 0.122	0.20
Disease duration, months	108 (48–192)	0.154	0.102
DAS-28	3.76 (2.80–4.51)	0.223	0.018
RF positivity, n (%)	97 (85.8)	0.018	0.85
ACPA positivity, n (%)	72 (63.7)	− 0.257	0.008
Ln-RHI	0.687 ± 0.329	− 0.221	0.019
CRP, mg/dL	0.366 (0.23–0.80)	0.132	0.16
ESR, mm/h	25 (13–39)	0.149	0.12
NLR	2.10 (1.60–2.88)	0.075	0.44
IMA, ABSU	0.748 (0.718–0.778)	–	–

Values are mean ± SD or median (IQR).

*ACPA* anticitrullinated cyclic peptide antibodies, *BMI* body mass index, *CRP* C-reactive protein, *DAS-28* disease activity score-28, *DMARDs* disease-modifying antirheumatic drugs, *ESR* erythrocyte sedimentation rate, *IMA* ischemic modified albumin, *Ln-RHI* log-transformed reactive hyperemia index, *NLR* neutrophils to lymphocytes ratio.

Despite not stated as a formal procedure in the protocol, when possible, a sex-balanced enrollment was performed with the aim of minimizing the effect of sex on measure of endothelial function and serum IMA concentrations.

Even if a large proportion of RA patients were under immunosuppressive drugs on enrollment (79/113, 70%), only a minority were in clinical remission (18/113, 10.6%) (Table 1). In line with a previous report^9^, ED, defined as an impaired peripheral vasodilatory capacity (Ln-RHI < 0.51)^25^, was present in about one-third of RA patients (30/113, 27%) (Table 1).

### IMA concentrations and RA disease activity

Mean serum IMA concentrations in the overall group of RA patients without previous cardiovascular events were 0.748 (0.718–0.778) ABSU.

**Figure 1:**
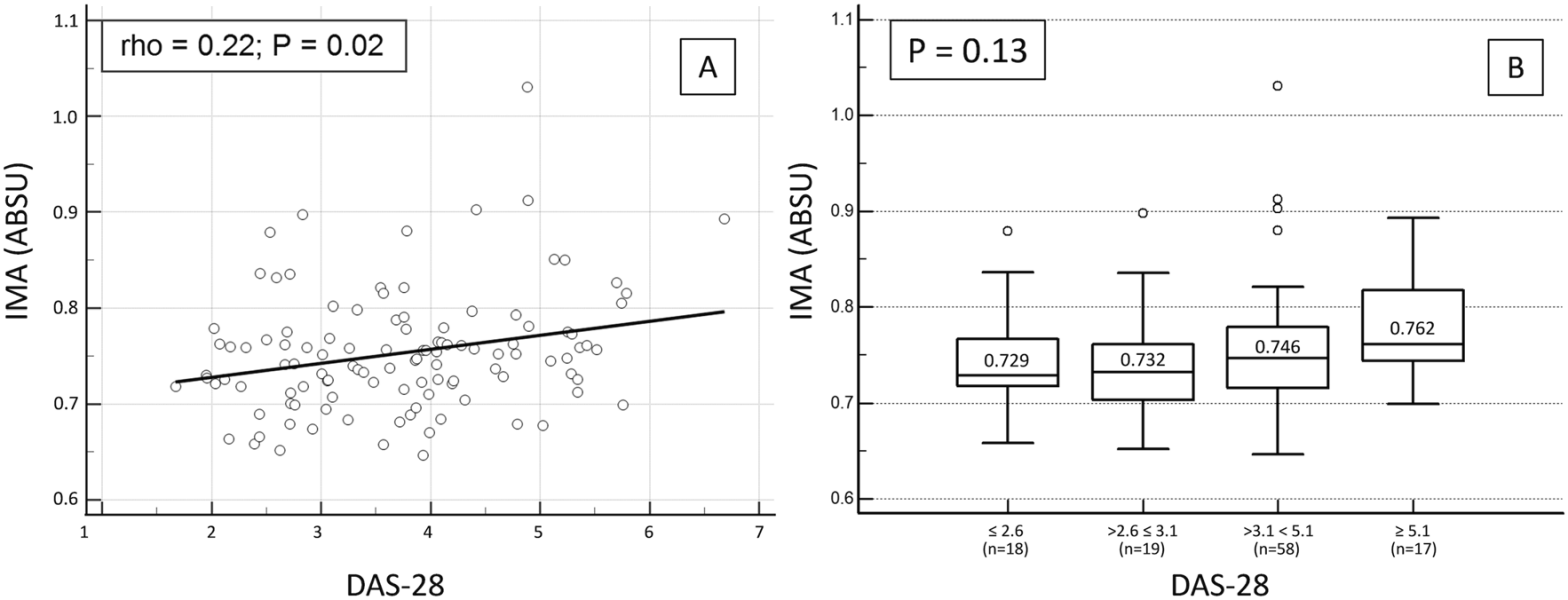
Correlations between serum IMA concentrations and RA disease activity.

IMA values were positively correlated with RA disease activity measured as DAS-28 (rho = 0.22, p = 0.02) (Table 1, Fig. 1A). However, there was only a trend towards a significant difference in serum IMA concentrations across DAS28-defined categories of increasing disease activity [median 0.729 (IQR 0.718–0.767) ABSU in the group in clinical remission; 0.732 (IQR 0.703–0.761) ABSU in RA patients with low disease activity; 0.746 (IQR 0.716–0.780) ABSU in RA patients with moderate disease activity; 0.762 (IQR 0.744–0.818) ABSU in RA patients with high disease activity; p = 0.52 (Fig. 1B).

Moreover, there was no significant association between DAS-28 and serum IMA concentrations in univariate logistic regression analysis [OR 24.1 (95% CI 0.003 to 178,257), p = 0.48].

### Relationship between serum IMA concentrations and peripheral vasodilatory capacity

**Figure 2:**
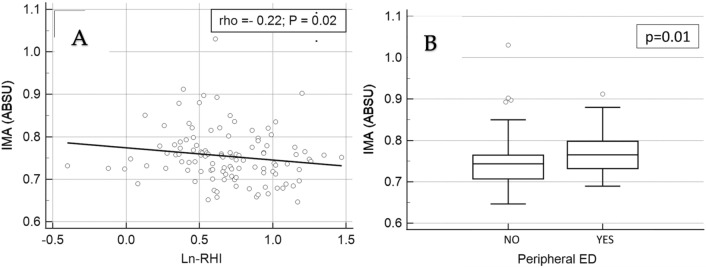
Correlations between serum IMA concentrations and peripheral vasodilatory capacity.

Peripheral vasodilatory capacity expressed as Ln-RHI showed a significant inverse correlation with serum IMA concentrations (rho =  − 0.22, p = 0.02) (Fig. 2A). Accordingly, IMA values were significantly increased in RA patients with ED (n = 30) when compared to those without (n = 83) [median 0.765(IQR 0.707–0.765) ABSU vs. 0.744 (IQR 0.732–0.798) ABSU, respectively; p = 0.01] (Fig. 2B).

Univariate logistic regression analysis (Table 2) confirmed a significant relationship between ED and serum IMA concentrations (OR 1173, 95% CI 1.36–1,013,645) and also demonstrated a significant association between ED and DAS-28 (OR 1.4899, 95% CI 1.0031 to 2.2129, p = 0.048). In multivariate logistic regression (Table 3), serum IMA concentrations remained significantly associated with ED after correction for confounders.

**Table 2:** Univariate logistic regression analysis assessing the association between patient characteristics and endothelial dysfunction.

ED	Crude OR	95% CI	p-value
Age	1.00	0.95 to 1.07	0.78
Gender	1.06	0.44 to 2.52	0.90
BMI	1.03	0.95 to 1.12	0.47
Smoking status	1.48	0.61 to 3.58	0.39
Dyslipidaemia	0.83	0.30 to 2.36	0.74
Hypertension	1.69	0.67 to 4.25	0.27
Diabetes	1.43	0.54 to 3.78	0.47
Steroid use	1.41	0.59 to 3.35	0.44
Metotrexate use	1.14	0.49 to 2.68	0.76
DMARDs use	1.30	0.51 to 3.31	0.58
Disease duration	1.00	0.99 to 1.00	0.68
DAS-28	1.49	1.00 to 2.21	**0.048**
RF	0.58	0.19 to 1.77	0.34
ACPA	0.51	0.22 to 1.23	0.14
CRP	0.99	0.94 to 1.04	0.70
ESR	1.02	0.10 to 1.04	0.059
NLR	1.27	0.88 to 1.84	0.19
IMA	1.17	1.36 to 10.13	**0.040**

*ACPA* anticitrullinated cyclic peptide antibodies, *BMI* body mass index, *CRP* C-reactive protein, *DAS-28* disease activity score-28, *DMARDs* disease-modifying antirheumatic drugs, *ESR* erythrocyte sedimentation rate, *IMA* ischemic modified albumin, *Ln-RHI* Log-transformed reactive hyperemia index, *NLR* neutrophils to lymphocytes ratio.

In bold significant values.

**Table 3:** Multivariate logistic regression analysis assessing the association between patient characteristics and peripheral endothelial dysfunction.

Peripheral endothelial dysfunction	OR	95% CI	p-value
Model 1			
DAS-28	0.41	0.90 to 2.22	0.14
ACPA	1.42	0.94 to 2.14	0.09
NLR	0.51	0.19 to 1.34	0.17
IMA	2.25	1.06 to 4787.50	**0.048**
Model 2			
ESR	1.02	0.9974 to 1.0392	0.09
ACPA	0.38	0.9143 to 2.0777	0.13
NLR	0.57	0.2221 to 1.4512	0.24
IMA	7.22	4.15 to 12,552.85	**0.02**

*BMI* body mass index, *CRP* C-reactive protein, *Ln-RHI* Log-transformed reactive hyperemia index, *NLR* neutrophils to lymphocytes ratio.

*ACPA* Anticitrullinated cyclic peptide antibodies, *DAS-28* disease activity score-28, *DMARDs* disease-modifying antirheumatic drugs, *ESR* erythrocyte sedimentation rate, *IMA* ischemic modified albumin, *NLR* neutrophils to lymphocytes ratio. Two models were created given the collinearity of DAS-28 and ESR. The models included all confounders showing associations with p < 0.2 in univariate logistic regression (ACPA, ESR, DAS28, IMA, NLR).

Significant values are in bold.

## Discussion

In this study, we reported for the first time, the presence of a significant relationship between serum IMA levels and impaired peripheral vasodilatory capacity in patients with RA, that appears to be independent from common atherosclerotic cardiovascular risk factors. A range of distinctive potential mechanisms at play in RA patients, including ED, atherosclerosis and microthrombosis, may be supposed to underly this association.

ED has been demonstrated early in the natural history of RA^26^ and seems to be related to chronic inflammatory burden, and increased prevalence of conventional cardiovascular risk factors with changes in lipid and glucose metabolism, metabolic syndrome, and side effect of specific immunosuppressive drugs having a crucial role^4,6,27^. Reduced peripheral vasodilatory capacity, may also depends on unbalanced autonomic nervous system control^9^ in RA patients with immuno-autonomic dysfunctions^28,29^. Autonomic changes of microvascular tone, may favor repetitive cycles of ischemia–reperfusion, leading in turn to tissue damage. Therefore, in the setting of reduced vasodilatory response, raised IMA levels may represent the result of chronic endothelial damage, ischemia, and increase oxidative stress. In accordance with this hypothesis, IMA levels have been reported to be correlated to ED, measured by flow-mediated dilatation (FMD), in male diabetic patients with erectile dysfunction^30^.

Atherosclerotic process in autoimmune diseases and RA, has been reported to develop early in arterial walls and may recognize multiple mechanisms including inflammatory response, local breach of tolerance, and macrophages migration and accumulation underneath the endothelial layer. Atherosclerotic plaque may decrease local blood flow inducing a condition of chronic tissue hypoxia and increased oxidative stress. Occlusion of coronary vessels is acutely followed by an increase of serum IMA concentration in patients with coronary syndrome, but it is likely that also chronic hypoxia in atherosclerotic arterial beds may be associated to raised serum IMA concentrations. Accordingly, Uslu et al.^19^ reported a significant correlation between serum IMA concentrations and carotid intima-media thickness in RA population.

Microthrombosis, linked to proinflammatory cytokines, pro-thrombotic autoantibodies^31^, and damaged endothelial barrier, may induce local tissue ischemia, oxidative stress and in increased albumin oxidation in RA. Accordingly, raised IMA levels have been reported in COVID-19, deep venous thrombosis, and pulmonary embolism^32,33^.

In our study there was only a trend towards a significant association between serum IMA levels and RA disease activity. Despite chronic inflammation would be crucial in the development of ED, microthrombosis and atherosclerosis, it is difficult to find an association between current indirect measures of inflammation and serum IMA levels. Most of our patients were under immunosuppressive treatment and therefore we cannot rule out the confounding effect of specific drugs on the relationship between IMA and peripheral vasodilatory capacity^34–37^.

Our study has some limitations. First, we did not prospectively investigate whether serum IMA concentrations predict ischemic events in our RA population. Second, measures of endothelial function with FMD (a gold-standard biomarker of ED) and other invasive techniques would have strengthen EndoPAT findings and should be implemented in future research on this subject. Third, the lack of a control group does not allow to establish whether the observed correlation between IMA and ED is specific to RA.

## Conclusions

Serum IMA concentrations are independently associated with impaired vasodilatory capacity and the presence of ED in RA patients free of overt cardiovascular disease. Further studies are needed to explore the mechanisms underlying this association and the potential role of IMA as a candidate biomarker for early atherosclerotic cardiovascular disease in RA.

## Data Availability

The data presented in this study are available on request from the corresponding author.

## References

[1] Gravallese EM, Firestein GS (2023). Rheumatoid arthritis—Common origins, divergent mechanisms. N. Engl. J. Med..

[2] Humphreys JH, Warner A, Chipping J, Marshall T, Lunt M, Symmons DPM, Verstappen SMM (2014). Mortality trends in patients with early rheumatoid arthritis over 20 years: Results from the Norfolk Arthritis Register. Arthritis Care Res. (Hoboken).

[3] del Rincón ID, Williams K, Stern MP, Freeman GL, Escalante A (2001). High incidence of cardiovascular events in a rheumatoid arthritis cohort not explained by traditional cardiac risk factors. Arthritis Rheum..

[4] Cacciapaglia F, Spinelli FR, Erre GL, Gremese E, Manfredi A, Piga M, Sakellariou G, Viapiana O, Atzeni F, Bartoloni E (2023). Italian recommendations for the assessment of cardiovascular risk in rheumatoid arthritis: A position paper of the cardiovascular obesity and rheumatic disease (CORDIS) study group of the Italian Society for Rheumatology. Clin. Exp. Rheumatol..

[5] Cacciapaglia F, Spinelli FR, Piga M, Erre GL, Sakellariou G, Manfredi A, Viapiana O, Fornaro M, Colella S, Floris A (2022). Estimated 10-year cardiovascular risk in a large Italian cohort of rheumatoid arthritis patients: Data from the cardiovascular obesity and rheumatic disease (CORDIS) study group. Eur. J. Intern. Med..

[6] Erre GL, Cacciapaglia F, Sakellariou G, Manfredi A, Bartoloni E, Viapiana O, Fornaro M, Cauli A, Mangoni AA, Woodman RJ (2022). C-reactive protein and 10-year cardiovascular risk in rheumatoid arthritis. Eur. J. Intern. Med..

[7] Erre GL, Bartoloni E, Viapiana O, Gremese E, Atzeni F (2023). “Cardiovascular, Obesity and Rheumatic Disease Study (CORDIS) Group” of the Italian Society of Rheumatology (SIR) C-reactive protein level association with future cardiovascular events assessed by different risk scores among rheumatoid arthritis patients. Eur. J. Intern. Med..

[8] Erre GL, Piras A, Mura S, Mundula N, Piras M, Taras L, Longu MG, Saba PS, Ganau A, Carru C (2016). Asymmetric dimethylarginine and arterial stiffness in patients with rheumatoid arthritis: A case-control study. J. Int. Med. Res..

[9] Erre GL, Piga M, Fedele AL, Mura S, Piras A, Cadoni ML, Cangemi I, Dessi M, Di Sante G, Tolusso B (2018). Prevalence and determinants of peripheral microvascular endothelial dysfunction in rheumatoid arthritis patients: A multicenter cross-sectional study. Mediat. Inflamm..

[10] Erre GL, Buscetta G, Paliogiannis P, Mangoni AA, Carru C, Passiu G, Zinellu A (2018). Coronary flow reserve in systemic rheumatic diseases: A systematic review and meta-analysis. Rheumatol. Int..

[11] Erre GL, Piras A, Piga M, Fedele AL, Mangoni AA, Lazzerini PE, Gremese E, Mathieu A, Ferraccioli G, Passiu G (2020). QT and QT dispersion intervals in long-standing and moderately active rheumatoid arthritis: Results from a multicentre cross-sectional study. Clin. Exp. Rheumatol..

[12] Bassu S, Zinellu A, Sotgia S, Mangoni AA, Floris A, Farina G, Passiu G, Carru C, Erre GL (2020). Oxidative stress biomarkers and peripheral endothelial dysfunction in rheumatoid arthritis: A monocentric cross-sectional case-control study. Molecules.

[13] Erre GL, Mangoni AA, Passiu G, Bassu S, Castagna F, Carru C, Piga M, Zinellu A, Sotgia S (2020). Comprehensive arginine metabolomics and peripheral vasodilatory capacity in rheumatoid arthritis: A monocentric cross-sectional study. Microvasc. Res..

[14] Mangoni AA, Tommasi S, Sotgia S, Zinellu A, Paliogiannis P, Piga M, Cauli A, Pintus G, Carru C, Erre GL (2021). Asymmetric dimethylarginine: A key player in the pathophysiology of endothelial dysfunction, vascular inflammation and atherosclerosis in rheumatoid arthritis?. Curr. Pharm. Des..

[15] Sanna GD, Piga M, Piga A, Falco O, Ponti E, Cauli A, Floris A, Mangoni AA, Casu G, De Luca G (2023). Prevalence and clinical significance of electrocardiographic signs of atrial myopathy in rheumatoid arthritis: Results from the EDRA study. Clin. Exp. Rheumatol..

[16] Mangoni AA, Zinellu A (2022). Serum concentrations of ischaemia-modified albumin in acute coronary syndrome: A systematic review and meta-analysis. J. Clin. Med..

[17] Shevtsova A, Gordiienko I, Tkachenko V, Ushakova G (2021). Ischemia-modified albumin: Origins and clinical implications. Dis. Mark..

[18] Balta S (2021). Endothelial dysfunction and inflammatory markers of vascular disease. Curr. Vasc. Pharmacol..

[19] Uslu AU, Kucuk A, Balta S, Ozturk C, Arslan S, Tekin L, Kucuksen S, Toker A, Kayrak M (2019). The relation between ischemia modified albumin levels and carotid intima media thickness in patients with rheumatoid arthritis. Int. J. Rheum. Dis..

[20] Ram Chander S, Varikasuvu SR, Anil Kumar P, Rupanagudi A (2017). Ischemia modified albumin concentrations in patients with rheumatoid arthritis. Int. J. Rheum. Dis..

[21] Leitemperguer MR, Tatsch E, Kober H, De Carvalho JAM, Moresco RN, Da Silva JEP (2014). Assessment of ischemia-modified albumin levels in patients with rheumatoid arthritis. Clin. Lab..

[22] Aletaha D, Neogi T, Silman AJ, Funovits J, Felson DT, Bingham CO, Birnbaum NS, Burmester GR, Bykerk VP, Cohen MD (2010). 2010 Rheumatoid arthritis classification criteria: An American College of Rheumatology/European League Against Rheumatism Collaborative Initiative. Arthritis Rheum..

[23] Erre GL, Paliogiannis P, Castagna F, Mangoni AA, Carru C, Passiu G, Zinellu A (2019). Meta-analysis of neutrophil-to-lymphocyte and platelet-to-lymphocyte ratio in rheumatoid arthritis. Eur. J. Clin. Investig..

[24] Erre GL, Buscetta G, Mangoni AA, Castagna F, Paliogiannis P, Oggiano M, Carru C, Passiu G, Zinellu A (2020). Diagnostic accuracy of different blood cells-derived indexes in rheumatoid arthritis: A cross-sectional study. Medicine (Baltimore).

[25] Bonetti PO, Pumper GM, Higano ST, Holmes DR, Kuvin JT, Lerman A (2004). Noninvasive identification of patients with early coronary atherosclerosis by assessment of digital reactive hyperemia. J. Am. Coll. Cardiol..

[26] Vaudo G, Marchesi S, Gerli R, Allegrucci R, Giordano A, Siepi D, Pirro M, Shoenfeld Y, Schillaci G, Mannarino E (2004). Endothelial dysfunction in young patients with rheumatoid arthritis and low disease activity. Ann. Rheum. Dis..

[27] Cacciapaglia F, Spinelli FR, Bartoloni E, Bugatti S, Erre GL, Fornaro M, Manfredi A, Piga M, Sakellariou G, Viapiana O (2023). Clinical features of diabetes mellitus on rheumatoid arthritis: Data from the cardiovascular obesity and rheumatic disease (CORDIS) study group. J. Clin. Med..

[28] Ingegnoli F, Buoli M, Antonucci F, Coletto LA, Esposito CM, Caporali R (2020). The link between autonomic nervous system and rheumatoid arthritis: From bench to bedside. Front. Med. (Lausanne).

[29] Taylor PC, Holman AJ (2019). Rheumatoid arthritis and the emergence of immuno-autonomics. Rheumatology (Oxford).

[30] Kurt HA, Demirci E, Alan C (2022). Endothelial dysfunction and ischemia-modified albumin levels in males with diabetic and nondiabetic erectile dysfunction. Dis. Mark..

[31] Erre GL, Bosincu L, Faedda R, Fenu P, Masala A, Sanna M, Taras L, Longu MG, Piras M, Soro G (2014). Antiphospholipid syndrome nephropathy (APSN) in patients with lupus nephritis: A retrospective clinical and renal pathology study. Rheumatol. Int..

[32] Saglam E, Sener G, Bayrak T, Bayrak A, Gorgulu N (2023). Analysis of ischemia-modified albumin (IMA) and coagulation parameters in patients with SARS-CoV-2 pneumonia. J. Clin. Med..

[33] Turedi S, Patan T, Gunduz A, Mentese A, Tekinbas C, Topbas M, Karahan SC, Yulug E, Turkmen S, Ucar U (2009). Ischemia-modified albumin in the diagnosis of pulmonary embolism: An experimental study. Am. J. Emerg. Med..

[34] Mangoni AA, Erre GL (2020). Repurposing available anti-inflammatory and immunomodulating agents for cardiovascular risk management: A call for submissions to current clinical pharmacology. Curr. Clin. Pharmacol..

[35] Mangoni AA, Tommasi S, Zinellu A, Sotgia S, Bassu S, Piga M, Erre GL, Carru C (2019). Methotrexate and vasculoprotection: Mechanistic insights and potential therapeutic applications in old age. Curr. Pharm. Des..

[36] Mangoni AA, Woodman RJ, Piga M, Cauli A, Fedele AL, Gremese E, Erre GL (2021). EDRA study group patterns of anti-inflammatory and immunomodulating drug usage and microvascular endothelial function in rheumatoid arthritis. Front. Cardiovasc. Med..

[37] Atzeni F, Rodríguez-Carrio J, Popa CD, Nurmohamed MT, Szűcs G, Szekanecz Z (2021). Cardiovascular effects of approved drugs for rheumatoid arthritis. Nat. Rev. Rheumatol..

